# Improving Precautionary Communication in the EMF Field? Effects of Making Messages Consistent and Explaining the Effectiveness of Precautions

**DOI:** 10.3390/ijerph13100992

**Published:** 2016-10-09

**Authors:** Christoph Boehmert, Peter Wiedemann, Rodney Croft

**Affiliations:** 1 Department of Science Communication, Karlsruhe Institute of Technology, 76131 Karlsruhe, Germany; 2 Australian Centre for Electromagnetic Bioeffects Research, University of Wollongong, Wollongong NSW 2522, Australia; rcroft@uow.edu.au; 3 School of Psychology, University of Wollongong, Wollongong NSW 2522, Australia; peter.wiedemann@mac.com; 4 Illawarra Health and Medical Research Institute, University of Wollongong, Wollongong NSW 2522, Australia

**Keywords:** precaution, EMF, risk communication, conditional risk perception

## Abstract

Many radiation health agencies communicate precautionary measures regarding the use of mobile communication devices, e.g. the use of a headset while talking on the phone. These precautionary messages have, however, been shown to unintentionally increase risk perceptions about radiofrequency electromagnetic fields (RF-EMFs). The current study tested two potential ways of amending precautionary messages in order to minimise this unintentional effect. Firstly, the messages’ potential to be perceived as inconsistent and thereby raise suspicions was addressed; secondly, the effectiveness of the precautions was explained. An experimental design was applied in which a quota sample of 1717 Australian residents was randomly assigned to one of six message conditions. Three different risk perception measures served as dependent variables, two of them are conditional measures. The original effect of precautionary messages to amplify risk perceptions could not be replicated. Furthermore, amending precautionary messages in favour of more consistency had no effect, while explaining the effectiveness of the precautions increased conditional risk perception under the condition that no precautions are taken. This was contrary to our assumptions. We infer from these results that changing precautionary messages in terms of consistency and effectiveness in order to reduce risk perception is hardly possible. The use of conditional risk perception measures seems fruitful for studies looking at the effects of precautionary or protective messages, given that previous studies have only investigated effects on unconditional risk perception. However, the present results should not be over-interpreted as the measures’ validity in the EMF context still needs further investigation.

## 1. Introduction

In radiation protection, the precautionary principle is a backbone of risk policy. In line with this maxim, precautionary messages regarding the use of mobile communication devices are disseminated by radiation health authorities in various countries, e.g. in Australia, Austria, France, Germany and the United Kingdom [1,2,3,4,5]. The term precaution itself can be defined in different ways; indeed there exist many different versions of the so called precautionary principle, some of them weaker and others stronger (see for example [6,7,8,9]). With the provision of precautionary messages, public health authorities aim at providing protection without unduly increasing concern. What is seen as a “reasonable amount” of concern is debatable for any kind of risk, of course. Nevertheless, when comparing proven health risks like smoking and uncertain risks like exposure to radiofrequency electromagnetic fields (RF-EMFs) used in mobile telecommunications, it is obvious that for the former, increasing risk perceptions seems a reasonable goal, while for the latter the benefit of increasing risk perceptions is less clear. Thus, the proportionality of public concern and the risk at hand should be kept in mind when informing about risks and precautions.

Yet desired effects and the effects that risk communication efforts factually have on their recipients are two different things. Mostly unexpected by radiation health authorities, precautionary communication regarding potential health effects of exposure to RF-EMFs used in mobile telecommunications has been shown to increase recipients’ risk perception about RF-EMFs [10,11,12,13,14]. This effect seems to be robust, as it has been shown with different kinds of messages and by different researchers. However, one study [15], using information brochures as stimuli, did not find an effect of precautionary messages. In that study, information about RF-EMFs and mobile phone and base station radiation patterns increased mobile phone risk perception, regardless of whether there were additional precautionary recommendations. Furthermore, limiting the generalizability of the effect, most of the studies reporting it have been conducted with ad-hoc [10,13] or student samples [11,12], a limitation that the current study intends to overcome.

Still, until recently, how precautionary messages increase recipients’ concerns about potential health effects has not been investigated in depth. A recent study found that the effect is dependent on recipient characteristics [16], but cognitive or affective processes involved in the effect remain unknown. Furthermore, and more interesting from a practical risk communication perspective, it is also unknown whether precautionary messages can be amended such that they do not unintentionally increase participants’ risk perception. The current study attempts to answer the latter question. The practical implications of this research are clear—if there is a way to amend precautionary recommendations so that they do not unintentionally increase participants’ risk perceptions, then these amendments should be applied to precautionary communication. If there is no way to reduce the concern-increasing effect of precautionary messages, then authorities need to reconsider their communication practice regarding, at least, the handling of public RF-EMF related concerns and worries. Of course, the above assumes that increasing concern is not a desired outcome of the precautionary message, but this may not always be the case. For example, heightened concern can be seen as a facilitating condition for applying precautionary behaviours (in line with Roger’s Protection Motivation Theory outlined below), and so if communicators want recipients to carry out the recommended protective behaviours, then the heightened concern would be a desired effect. However, for the purpose of this paper, in relation to RF-EMFs, it is assumed that the goal is not to “increase concern in order to encourage protective behaviours”.

### 1.1. Two Potential Ways to Alter Precautionary Messages

#### 1.1.1. Inconsistency of Precautionary Messages

From our perspective, there are two conceivable reasons for the countervailing concern-increasing effect of precautionary communication. The first one is inherent to the logic of any precautionary message. Precautionary messages inform about already implemented precautions (e.g. by a government) or about precautions that should be implemented in the future (e.g. by the message recipient) due to potential health risks. That is, precautionary messages do, either implicitly or explicitly, suggest a health risk. The second reason has to do with the communication of uncertainty. As Longman et al. (2012) [17] have shown, communicating uncertainty results in increased risk perceptions and decreased perceived credibility. Communicating precautionary messages may, therefore, undermine the trust established in exposure guidelines, as already feared by the WHO in 2000 [18]. That is, it is not necessarily logical for people to believe both that the guidelines protect from detrimental health effects, and that precautionary measures are needed. Past qualitative research [19] suggests that message recipients notice and interpret this inconsistency. In that focus group study, participants mentioned that “it’s quite contradictory to say that there are none [no evidence of risk] and then to recommend caution”. Timotijevic and Barnett [19] also reported participants voicing their suspicions (“I’m sure they know more than they’re letting on”). Although the study provides no explicit link between the perceptions of inconsistency and these suspicions, it seems plausible that the former gives rise to the latter.

Wiedemann, Boerner and Claus (2016) [20] propose a theoretical explanation for the role of inconsistency in the effect of precautionary messages. They assume that the inconsistency of the message results in a state of cognitive dissonance [21], and that this dissonance amplifies one’s risk perception. This dissonance can be reduced by questioning the claim that the existing limit values for RF-EMF exposure protect public health, or by questioning the claim that precautionary measures are needed. The questioning of the first claim should result in increasing one’s risk perception.

In the current study, we address this issue by disclosing a potential rationale behind the communication of the precautionary measures so as to reduce the perception of inconsistency (i.e. providing concerned people with a measure to reduce their exposure to RF-EMFs should they wish to reduce it, rather than it being recommended because the science shows that it will reduce risk; see Appendix A for the wording). Our hypothesis pertaining to message consistency is that reducing the precautionary message’s potential to be perceived as inconsistent will reduce risk perception, compared to a precautionary message without the text to reduce the apparent inconsistency.

#### 1.1.2. Belief in the Effectiveness of Precautionary Measures

A classical theory of health behaviour is protection motivation theory (PMT) [22,23]. The PMT was developed to investigate the effects of a specific kind of health risk message: fear appeals. The theory was developed by Ronald W. Rogers who was the first to postulate three essential components of a fear appeal, namely: (1) the probability that a threat would occur; (2) its magnitude of noxiousness; and (3) the effectiveness of the recommended response [22]. These message components are able to influence the central cognitive constructs that in turn influence protection motivation. In a revised model [23], the central cognitive constructs of PMT, a threat appraisal and a coping appraisal, are each determined by several variables. Threat appraisal is determined by the perceived severity of a threat, the perceived vulnerability to that threat and intrinsic or extrinsic rewards of a maladaptive behaviour. The coping appraisal is determined by the perceived efficacy of a (protective) behaviour, a person’s self-efficacy and the response costs.

While PMT tries to predict protective behaviour or behavioural intentions, our main variable of interest in the context of precautions against the potential health effects of exposure to RF-EMFs is risk perception. Hence, we suggest an effect that has not been modelled in PMT—namely a direct relation between perceived efficacy and risk perception (or “threat appraisal” as it is termed in PMT). Positing this relationship seems generally plausible, given that the relation between efficacy appraisal and threat appraisal has been a major point of discussion in PMT [24] (see also the paragraph about conditional risk perception measurement below). Further, it seems a reasonable approach for investigating the effects of RF-EMF precautionary information specifically.

In order to understand how PMT may be relevant in the case of RF-EMFs from mobile communication devices, it is important to note that there are some common misconceptions among lay people regarding RF-EMF exposure patterns [25]: (1) exposure is erroneously believed to decrease linearly, and not with the inverse square of the distance from the source; and (2) the relative contribution of base stations to overall exposure is overestimated, and hence the contribution of mobile phones underestimated. Consistent with this view, concerns about RF-EMFs emitted by base stations are higher than concerns about RF-EMFs from mobile phones [12,16,25,26]. In fact, many people do not appreciate that their mobile phone itself is a small base station [25]. Thus, it would be expected that recipients underestimate the effectiveness of precautionary measures communicated by radiation health authorities because these aim at increasing a person’s distance to the mobile phone or at reducing the strength of the RF-EMF emitted by the phone. Our hypothesis pertaining to the effectiveness of the precautions thus states that emphasizing the effectiveness of precautionary measures by: (1) explaining the importance of the distance variable; and (2) explaining that mobile phones are responsible for a larger share of personal exposure than base stations, would attenuate the precautionary message’s potential to increase risk perception.

Claassen et al. (2015) [27] conducted a study providing their participants with similar information. One of their text modules explained the distance-exposure relationship and also the relative contribution of nearby EMF sources (e.g. mobile phones) versus sources that are far away (e.g. base stations). In contrast to the current study, Claassen et al. [27] focused on mere knowledge provision and did not use these facts to explain the relative effectiveness of precautionary measures. They did not find an effect of this text module on risk perception. However, given the rather small effect sizes of information provision on risk perception reported in previous studies [12,16], this study might have been underpowered with an N = 245 and a 2 × 2 × 2 group design. Furthermore, we think that communicating this information in order to explain the effectiveness is likely to change its effect.

### 1.2. A Methodological Issue: Conditional Risk Perception Measurement in Studies Testing Communications of Precautionary or Protective Measures

Risk perception is usually measured with one or more items that simply ask participants how risky, dangerous or threatening a particular situation or behaviour is. According to Ronis (1992) [28] and van der Velde et al. (1996) [29] this type of measurement captures people’s unconditional risk perception. “Unconditional” refers to the fact that it remains unknown if participants include the possibility of taking precautionary or protective actions in their rating. Opposed to this, van der Velde et al. [29] suggest that measures of conditional risk perception should be applied more often. Conditional risk perception measures are more closely related to the concepts used in various social cognitive models [24,28,30,31], among them the Protection Motivation Theory. An item capturing conditional risk perception either explicitly asks for the risk estimate “without precautions” (e.g. “How dangerous do you think the electromagnetic fields from mobile phones are while talking on the phone without using any precautionary measures?”) or “if precautions are taken” (e.g. “How dangerous do you think the electromagnetic fields from mobile phones are while talking on the phone if precautionary measures that you deem appropriate are used?”). We think that this distinction is very important in research studying the effects of the communication of precautionary or protective measures (i.e. the communication of measures against a proven risk) as an unconditional measurement is not able to tap potentially important processes. For instance, one person could think that exposure to RF-EMFs is very risky if no precautionary measures are taken (high conditional risk 1), but because she or he also thinks that there are effective precautionary measures at hand (low conditional risk 2), he or she may judge the unconditional risk as low. Another person might not include their view of conditional risk 2 in his or her rating of the unconditional risk and hence give a high risk estimate. This example shows that it can be quite difficult to interpret unconditional risk perception measures. Notably, all studies that have looked at the effects of precautionary messages about RF-EMFs have so far used unconditional risk perception measures [10,11,12,13,14,15,16].

Thus, in general, it is difficult to disentangle whether unconditional risk perceptions “drive health-protective intentions or whether these intentions are used to infer risk perceptions” [24]. To get a clearer picture of participants’ actual risk perceptions, we will therefore analyse conditional risk perception measures in the current study. To ensure the comparability with former studies, we will also analyse unconditional measures and compare these with the conditional measures. In the following hypotheses section every hypothesis is stated specifically for each of the three risk perception measures (unconditional risk perception, conditional risk perception without precautions and conditional risk perception if precautions are taken).

### 1.3. Hypotheses

**Hypothesis 1 (replication)**: In line with previous research, we hypothesise that risk perception will be higher after reading a precautionary message than after a basic message which does not contain precautionary information. The effect should be observed for unconditional risk perception (UR) and conditional risk perception without precautions (CR1). We do not expect to find the effect for conditional risk perception if precautions are taken (CR2). In fact, new knowledge about the measures might even cause CR2 to be lower after the precautionary message. As previous studies used UR measures, the replication with the unconditional measure (hypothesis 1a) can be regarded as a “literal replication” according to the terminology of replications introduced by Kelly, Chase and Tucker (1979) [32]. Hypotheses 1b and 1c test the effect of the same experimental variation on dependent variables that have previously not been used. In the terminology of Kelly et al. [32], this can be called an operational replication.

Hypothesis 1a: UR_precaution_ > UR_basic message_

Hypothesis 1b: CR1_precaution_ > CR1_basic message_

Hypothesis 1c: CR2_precaution_ ≤ CR2_basic message_

The previous studies on the effects of precautionary messages were based on a simple factorial design. Here, the decisive factor referred to precaution, and, usually, two experimental conditions are used: a basic text about how the exposure guidelines protect health, and a precautionary text that informs about additional precautionary measures [12]. Following the basic concept of the Solomon four group design, it seems useful to add a further treatment step. It consists of a group that receives no information at all about RF-EMFs in mobile telecommunications. This allows one to test for an effect of the basic message stating the safety of the current exposure limits. Such an effect is plausible on the basis of former studies (cf. [33]) and useful to investigate as, to our knowledge, this “safety” basic message forms part of many risk communication efforts by national health authorities.

**Hypothesis 2 (no message baseline)**: For the comparison of the basic message with the condition of not reading any text at all, we expect the same pattern as for the comparison of the precautionary message with the basic message UR and CR1. We expect the basic message also to increase CR2.

Hypothesis 2a: UR_basic message_ > UR_no message_

Hypothesis 2b: CR1_basic message_ > CR1_no message_

Hypothesis 2c: CR2 _basic message_ > CR2_no message_

**Hypothesis 3 (consistency)**: Regarding the consistency amendment of the precautionary message, we hypothesise that risk perception will be lower after the consistent precautionary message than after the original precautionary message. This effect should be observed for unconditional risk perception (UR) and conditional risk perception without precautions (CR1). It was not quite clear to us as to how increased message consistency might influence conditional risk perception if precautions are taken (CR2). We therefore hypothesise that we will not find an effect.

Hypothesis 3a: UR_precaution_ > UR_consistent precaution_

Hypothesis 3b: CR1_precaution_ > CR1_consistent precaution_

Hypothesis 3c: CR2_precaution_ = CR2_consistent precaution_

**Hypothesis 4 (effectiveness)**: Regarding the effectiveness amendment of the precautionary message, we hypothesise that risk perception will be lower after the precautionary message containing effectiveness information than after the original precautionary message. This effect should be observed for unconditional risk perception (UR) and conditional risk perception if precautions are applied (CR2). However, for the judgement of conditional risk perception, if precautions are not applied (CR1), the effectiveness amendment should not make any difference.

Hypothesis 4a: UR_precaution_ > UR_effective precaution_

Hypothesis 4b: CR1_precaution_ = CR1_effective precaution_

Hypothesis 4c: CR2_precaution_ > CR2_effective precaution_

Hypotheses 3 and 4 concern the specific effects of two additional text modules. However, in practice, the two types of information might be communicated together. Communicating both the consistency and effectiveness information may be useful to ensure that, if different recipients find different types of additional information important, then in this case all recipients would avoid unduly increasing their concern. Additionally, studying the effect of the combination of both text modules allows us to test the hypothesised pattern of influence of each text on each of the conditional variables once more. That is, while the effectiveness is assumed to influence CR2 but not CR1, consistency is hypothesised to act conversely.

**Hypothesis 5 (consistency plus effectiveness)**: We hypothesise that the combination of the consistency and effectiveness information will lead to decreased scores on all three risk perception variables, compared with each of the consistency and effectiveness conditions separately. The exceptions are that the additional effectiveness information is not hypothesised to influence CR1 and that the additional consistency information is not assumed to influence CR2.

Hypothesis 5a:(1) UR_effective + consistent precaution_ < UR_consistent precaution_ and
(2) UR_effective + consistent precaution_ < UR_effective precaution_Hypothesis 5b:(1) CR1_effective + consistent precaution_ = CR1_consistent precaution_ and
(2) CR1_effective + consistent precaution_ < CR1_effective precaution_Hypothesis 5c:(1) CR2_effective + consistent precaution_ < CR2_consistent precaution_ and
(2) CR2_effective + consistent precaution_ = CR2_effective precaution_

## 2. Materials and Methods

### 2.1. Sample

The study was conducted with an online panel of Australian residents managed by a global market research institute (Survey Sampling International). In order to increase the representativeness of the sample for the general population, non-interlocking quotas for gender, age and region of residence were applied for each experimental group separately (this procedure resulted in many “overquota responses”). In total, 2276 participants aged between 18 and 65 fully completed the study materials. Participants were excluded in three subsequent steps. Firstly, 181 participants were excluded because they had “straight lined” 20 questions on one page in a row. Secondly, 176 participants who had given a wrong answer to the question “what day was it yesterday” were excluded. Subsequently, 175 participants were discarded prior to the analyses because they had completed the experiment implausibly quickly, indicating that they did not read the text material and/or answered the questions without reading them properly. Pretesting in our lab had shown that completing only the questionnaire (no text condition, see Table 1) could not be done adequately in less than 5 min and 30 s. Conservatively, we excluded participants in this group if they had completion times <5 min. For the other groups, the completion time below which we excluded participants varied with the length of text they were presented with. For instance, Group 6 received all the text modules (see Appendix A). In this group, we excluded participants that had completion times <7 min. In addition, 27 participants were excluded because they had completion times >90 min (none of the results changed in terms of statistical significance if participants with completion times >90 min were included). The final sample consisted of 1717 participants. Participants received reward points from the market research company for their participation. These reward points could be combined with reward points from other studies and used to purchase vouchers, for example for online shops.

### 2.2. Procedure

Members of the market research company’s panel were contacted via email and invited to “take a survey”. The invitations randomly contained one of six links, allocating participants to one of the six experimental groups. If a person agreed to take the survey, they were then redirected to the start page of the survey that first checked their eligibility in terms of the above-mentioned quotas. If eligible, they were then provided with a participant information sheet informing them about the researchers, the aim of the research, their task and other general information. After the participant information sheet, they were provided with the different types of information. Five groups each received one of five versions of a message about exposure to RF-EMFs from mobile phones and health. One group did not receive any information and directly started with the questionnaire. The questionnaire was the same for all groups. After completing the questionnaire, participants were informed about the scope of the study and provided with the researcher contact information once again as well as with a link to the Australian Radiation Protection and Nuclear Safety Agency (ARPANSA), in case they wanted more information. The study was approved by the University of Wollongong Human Research Ethics Committee (Code: HE15/286).

### 2.3. Design and Information Provision

The six experimental conditions are listed in Table 1. The wording of the text modules can be found in Appendix A. A full 2 (basic text) × 2 (precaution) × 2 (consistency) × 2 (effectiveness) factorial design was not applicable as, for example, trying to make the message consistent by explaining why precaution is recommended is only meaningful if the precautionary information is actually given (and not only the basic message). Group 1, the “no text group” did not receive any information at all and started answering the questionnaire right away. Group 2, the “basic group” read a basic text about the safety of the current exposure limits. Group 3, the “precaution group” read the basic text and additionally a precautionary message about several ways to reduce personal exposure (e.g. by using a headset or a landline phone). Group 4, the “consistency group”, received the same information as the “precaution group” and additionally a short paragraph explaining the motives behind the communication of the precautionary measures. Group 5, the “effectiveness group”, received the same information as the “precaution group” and additionally a longer paragraph explaining the effectiveness of the precautionary measures, including a table. Group 6, the “consistency + effectiveness group” was provided with both the paragraphs about the motives for communicating precautionary measures and the effectiveness of the precautionary measures. The order of the text modules was always kept the same across all groups: (1) Basic text (2) precautionary text (3) consistency text (4) effectiveness text. In giving the basic text prior to the precautionary text, we follow the communication practice of national radiation health authorities, for instance those in Australia, France, Germany, Israel and the United States [3,4,5,34,35].

### 2.4. Questionnaire

**Figure 1:**
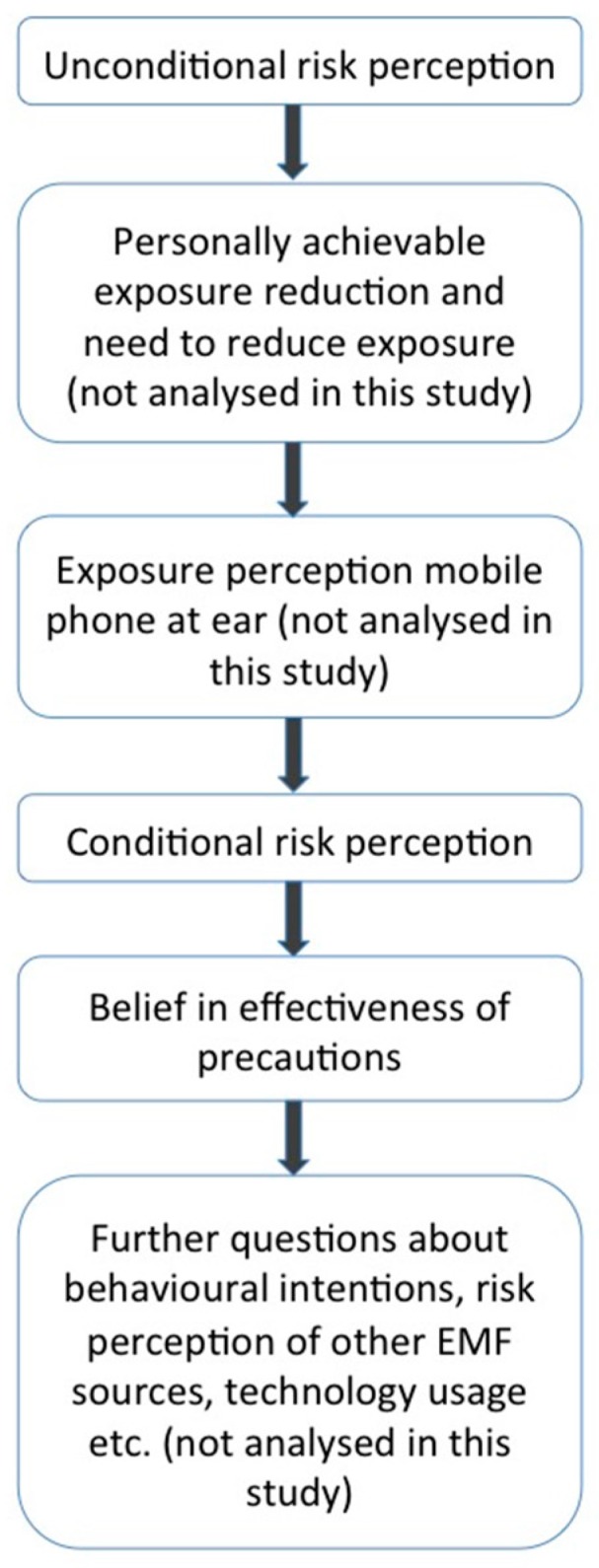
Flowchart of the course of the questionnaire.

The course of the questionnaire is summarised in Figure 1. The questionnaire started with the question for unconditional risk perception (“How dangerous do you think the electromagnetic fields from mobile phones are while you talk on the phone?”), followed by a question about their perceived control of mobile phone EMFs and a question about whether they think there is a need to reduce exposure to EMFs from mobile phones. On the next page, exposure perception was assessed by showing participants a picture of a man talking on a mobile phone held at his ear and asking them “In your opinion, how strong is the exposure to the person in the picture above?” On the next page, conditional risk perception without precautions (“How dangerous do you think the electromagnetic fields from mobile phones are while talking on the phone without using any precautionary measures?”) and conditional risk perception with precautions (“How dangerous do you think the electromagnetic fields from mobile phones are while talking on the phone if precautionary measures that you deem appropriate are used?”) were assessed.

The three risk perception items were all rated on seven-point Likert-scales that were numbered from one to seven and had labelled endpoints (1 = not dangerous at all, 7 = very dangerous). Previous studies of EMF risk perception have frequently used this type of scale ranging from no danger/risk to high danger/risk (e.g. [12,15,33,36,37]). Still, one has to be aware of the fact that the scale is imbalanced as all but one scale point indicate (to some extent) a danger/risk. Hence, this form of measurement can potentially overestimate “real” risk perception [38]. However, the absolute value of risk perception was of minor importance for the current study, as we were interested in the differences between the experimental groups.

Perceived control of exposure, need for exposure reduction and exposure perception were not analysed for the current study. On the subsequent page, participants indicated their belief in the effectiveness of six precautionary measures. On top of this page, they were presented with the initial statement “I believe that using the following measures reduces the likelihood of any negative health effects that mobile phone calls could have.” Below this statement they were given six precautionary measures (e.g. “Using a headset when making mobile phone calls”) and had to indicate the strength of their belief on a numbered Likert-scale ranging from “1 = I do not believe this at all” to “7 = I believe this very much”.

#### Order of Risk Perception Items

As described above, unconditional risk perception was assessed prior to the conditional risk perception questions and on a separate page. The unconditional item had to be assessed first because once the distinction between conditional risk perception without precautions and conditional risk perception with precautions is introduced, the lack of specification in the unconditional item becomes obvious and potentially confusing for the participants. The conditional risk perception items were both asked on the same page. Hence, participants could compare and adjust their answers relative to each other. How the two items interact with each other and if they would be answered differently if asked separately, are open questions. To mitigate potential unwanted effects, they were introduced with the following statement: “One of the previous questions was “How dangerous do you think the electromagnetic fields from mobile phones are while you talk on the phone”. We will now ask two related questions that focus on different aspects of your opinion. Some people might answer both questions differently while others might give the same answer. Please remember that we are interested in your opinion, so there is no right or wrong”. This statement was supposed to: (1) differentiate the items clearly from the unconditional item; and (2) avoid that the distinction between the two conditional items causes respondents to answer the questions differently “just because it seems logical to give different answers”. The order of the two questions was not varied and conditional risk perception without precautions was asked first. This order has been used previously [30], though it has to be noted that participants had to answer other questions in between the conditional items in the cited study. Furthermore, to us, it seems more in line with everyday thinking for the participants to rate the risk if nothing is done as a kind of baseline first and then to rate the risk if precautions are taken than doing it conversely.

### 2.5. Statistical Analyses

#### 2.5.1. Risk Perception Measurement

In order to obtain data on the characteristics of the three dependent variables (unconditional risk perception, conditional risk perception without precautions and conditional risk perception if precautions are taken), means and standard deviations were calculated for these variables on the level of the entire sample. The significance of the mean differences between the three variables was calculated using paired-samples *t*-tests. Pearson correlations were also calculated for these three variables.

#### 2.5.2. Effects of Different Types of Information on Risk Perception

For the analysis of the effects of different types of information on risk perception, we divided our hypotheses into two sets. The first set comprises hypotheses 1 and 2, which aim to replicate former results and analyse the effect of the basic message itself. Hypotheses 3 to 5 have a different scope. Each of these hypotheses aims to test the effect of a textual addition to the precautionary message. Therefore, we treat these hypotheses as the second set. For the two sets, separate one-way ANOVAs were calculated (see Table 2). For each of the three dependent variables (unconditional risk perception, conditional risk perception without precautions, and conditional risk perception with precautions) we conducted separate ANOVAs, resulting in six ANOVAs being calculated in total. In case of a significant ANOVA, we calculated independent samples *t*-tests to determine whether the hypothesised differences were observed. We used *t*-tests and not typical post-hoc tests like the Scheffé test or Tukey because we hypothesised specific differences a priori. In sum, we planned to conduct 18 *t*-tests (three for each of hypotheses one to four and six for hypothesis five). Type one error accumulation can be a problem when conducting multiple tests regarding the same hypothesis. In our case, however, hypotheses differ from each other and do not simply test the same effect on a different dependent variable. Nevertheless, we set α = 0.01 for the *t*-tests. For significant group differences, Cohen´s d was calculated as a measure of the effect size.

## 3. Results

### 3.1. Risk Perception Measurement

The means, standard deviations and correlations of the three risk perception variables for the whole sample are depicted in Table 3. As expected, the means differ significantly from each other (all *t* ≥ 15.57, all *p* < 0.001), with the mean of conditional risk perception without precautions (CR1) being higher than the mean of unconditional risk perception (UR). The mean of conditional risk perception with precautions (CR2) is much lower than both of the other means. All variables are highly and positively correlated.

### 3.2. Manipulation Check

In order to examine whether reading the text module about the precautionary measures’ effectiveness increased the belief in the effectiveness of the measures, the mean score of the six items capturing the beliefs in the different measures was calculated. Using an independent samples *t*-test, this mean score was then compared between the precaution group and the precaution plus effectiveness group. We did not include items for checking whether the consistency text module actually reduces perceptions of inconsistency. This is discussed as a limitation in the discussion section.

The assumptions for conducting the independent samples *t*-test for the comparison of the belief in the efficacy of the precautions were met (Levene’s test F = 0.493, *p* = 0.48, the Kolmogorov–Smirnov test indicated the deviation from the normal distribution in the groups but this can be neglected for large samples). The mean response effectiveness scores were 4.64 in the “precaution group” and 5.06 in the “effectiveness group”. This difference was highly significant (*t* = −4.055, *p* < 0.001) and indicates that the effectiveness text module served the intended function.

### 3.3. Effects of Different Types of Information on Risk Perception

Means and 99% confidence intervals of the three risk perception variables in each group can be found in Table 4. Table 5 summarises the outcomes of the hypothesis tests reported below.

#### 3.3.1. Hypotheses 1 and 2

A Levene’s test indicated that the homogeneity of variances assumption for calculating an ANOVA was not violated for any of the three dependent variables (all F < 2.065, all *p* > 0.12). A Kolmogorov–Smirnov test for normality of the distribution of errors in each group was significant for all three dependent variables, indicating the deviation from the normal distribution (all d > 0.191, all *p* < 0.001). These deviations were due to moderate negative kurtosis (−0.65 ≤ k ≤ 0.09 for all kurtosis values) and due to moderate skewness (−0.38 ≤ s ≤ 0.42 for all skewness values). While skewness does not influence ANOVA significance test, a negative kurtosis can inflate type one error [39,40]. However, because ANOVA is robust towards violations of the normality assumption for large samples, the kurtosis violation was moderate and because we adjusted for type 1 error for the subsequent *t*-tests, we proceeded with the ANOVA.

None of the three ANOVAs conducted yielded a significant result (for UR F = 2.742, *p* = 0.07; for CR1 F = 2.083, *p* = 0.13; for CR2 F = 0.516, *p* = 0.60). Thus, on all three risk perception variables, there were no differences among the “no text group”, the “basic group” and the “precaution group”. Hypotheses 1a and 1b and Hypotheses 2a, 2b and 2c are therefore rejected. The results are consistent with Hypothesis 1c.

#### 3.3.2. Hypotheses 3, 4 and 5

A Levene’s test indicated that the homogeneity of variances assumption for calculating an ANOVA was not violated for any of the three dependent variables (all F < 0.586, all *p* > 0.62). A Kolmogorov-Smirnoff test for normality of the distribution of errors in each group was significant for all three dependent variables, indicating the deviation from the normal distribution (all d > 0.187, all *p* < 0.001). These deviations were due to skewness (−0.81 ≤ s ≤ 0.57 for all skewness values) and moderate, mostly negative kurtosis (−0.50 ≤ k ≤ 0.38 for all kurtosis values). Due to the same reasoning as for the ANOVAs for Hypothesis 1 and 2, we still conducted the ANOVAs.

The ANOVA F-test showed significant group differences in UR (F = 3.142, *p* = 0.02) and in CR1 (F = 11.695, *p* < 0.001), but not in CR2 (F = 0.336, *p* = 0.799). This indicates that at least two of the included groups (precaution group, consistency group, effectiveness group and consistency + effectiveness group) differ significantly in the *t*-test from each other in UR and CR1 on a significance level of α = 0.05. For the significant ANOVAs, we proceeded with our planned comparisons using *t*-tests. As the ANOVA was not significant for CR2, we do not report any further *t*-tests for this variable. As CR2 group differences in the ANOVA were not significant, we rejected Hypotheses 4c and 5c(1). Finding no difference is consistent with Hypotheses 3c and 5c(2).

The homogeneity assumption was met for every *t*-test we calculated (all F < 1.205, all *p* > 0.27). The variables were not normally distributed, however, for group sizes of *n*_i_ > 30 the *t*-test is robust against a violation of this assumption.

##### 3.3.2.1. Pairwise Comparisons for Hypothesis 3

All mean differences between the “precaution group” and the “consistency group” were statistically not significant (*t_df_*_= 574_ = 1.345, *p* = 0.18 for UR and *t_df_*_= 574_ = 0.458, *p* = 0.65 for CR1). Hypotheses 3a and 3b therefore have to be rejected.

##### 3.3.2.2. Pairwise Comparisons for Hypothesis 4

The mean difference in UR between the “precaution group” and the “effectiveness group” was not significant (*t_df_*_= 569_ = −1.309, *p* = 0.19). Hypothesis 4a was therefore rejected. There was a highly significant difference between the two groups in CR1 (*t_df_*_= 569_ = −4.052, *p* < 0.001, Cohen´s d = 0.33), with the mean in the “effectiveness group” being higher. We hypothesised CR1 to be equal in the “precaution group” and the “effectiveness group”. Therefore, Hypothesis 4b was rejected.

##### 3.3.2.3. Pairwise Comparisons for Hypothesis 5

The “consistency group” and the “consistency + effectiveness group” differed considerably in UR, but not significantly on the applied significance level of α = 0.01 (*t_df_*_= 560_ = −2.536, *p* = 0.01). The “effectiveness group” and the “consistency + effectiveness group” did not differ in UR (*t_df_*_= 555_ = 0.077, *p* = 0.94). Therefore, Hypotheses 5a(1) and 5a(2) are rejected. For CR1, there was a significant difference between the “consistency group” and the “consistency + effectiveness group” (*t_df_*_= 560_ = −4.262, *p* < 0.001, Cohen´s d = 0.36) with the mean being higher in the “consistency + effectiveness group”. As we assumed the means to be equal, we rejected hypothesis 5b(1). The “effectiveness group” and the “consistency + effectiveness group” did not differ in CR1 (*t_df_*_= 555_ = 0.224, *p* = 0.82). Hypothesis 5b(2) was thus rejected.

## 4. Discussion

This study extended previous research that showed that precautionary messages about radiofrequency electromagnetic fields (RF-EMFs) increased recipient’s risk perceptions. Two ways of amending precautionary messages were tested. Firstly, an additional text module that addressed the inconsistency of precautionary messages being communicated along with messages stating the safety of the current exposure limits. Secondly, an additional text module that explained the effectiveness of the recommended measures was tested. Furthermore, this study introduced a new component to the measurement of risk perception in the field of precautions against EMFs. That is, while in previous studies risk perception had always been measured unconditionally, we additionally assessed two items attempting to capture participants conditional risk perceptions (i.e. the perception of the risk “if precautions are taken” and “if precautions are not taken”). Each of the items asked specifically for the risk “while talking on the phone”.

Before interpreting the results of the new message components, it has to be noted that the previously-reported effect of precautionary messages, an increase in risk perception, could not be replicated in the current study. Compared to a basic massage stating the safety of the existing limits, the addition of a precautionary message did not increase risk perceptions. Thus, in retrospect, the rationale of “avoiding the effect of precautionary messages by changing the message” that we formulated on the basis of existing research, does not fit the data we obtained. Why did we not find the previously reported effect of precautionary messages? There are several potential explanations for this. Firstly, risk perception in general might have gone down as mobile phone RF-EMF and health might not be considered as much an issue as was the case previously. However, this does not seem to be the case as the mean in the basic message group is similar to those of former studies (2.86 for the Australian subsample in Wiedemann et al. 2013 [12] and 2.91 in the current study; however, it is difficult to compare these two values as: (1) we had to transform the values of the current study from a seven-point to a five-point scale which can be problematic; and (2) the wording of the items was not exactly the same). Secondly, another explanation would be that the effect was sensitive to our change in what the risk perception question was specifically referring to. While in former studies risk perception was assessed for mobile phones in general, we assessed it for a specific exposure situation, namely “while talking on the phone”. Thirdly, while the current study was conducted with a general population sample, former studies have mostly tested student populations [11,12,16]. The effect might only exist in student samples. Nevertheless, analysing both new experimental conditions of “consistency” and “effectiveness” is still useful, especially from a practical risk communication point-of-view for which clear, consistent and effective messages are of special importance.

Increasing the message’s consistency by providing a rationale behind the communication of the precautionary information did not decrease risk perceptions significantly. That is, explaining that precautionary messages are only intended to provide behavioural options to those who are already concerned about potential health effects does not lower recipient’s risk perceptions. Apparently, perceptions of inconsistency are not important in the reception of precautionary messages. However, a limitation of our study is that we did not test whether our consistency text module actually was perceived as more consistent by the participants. An alternative explanation for our results is hence that the way in which we addressed the inconsistency simply did not lower the perceived inconsistency of the recipients. A further explanation is that people might apply a simple social heuristic “fear risks that are feared by other people” for their risk perceptions [41]: If some people are concerned about RF-EMF exposure then be cautious too.

We expected the additional text module that explained the effectiveness of the precautions to decrease conditional risk perception if precautions are taken and potentially also unconditional risk perception, compared to the precautionary message only. We found an opposing picture that, however, seems reasonable from a psychological view (see below). In response to the effectiveness text module people judged RF-EMFs from mobile phones as even more dangerous—but only if they judged the risk under the condition that no precautions are taken. Notably, the combination of both effectiveness and consistency information, which was tested as well, had the same effect. We see this as a sign of the robustness of the effect of communicating the measures’ effectiveness, and the lack of importance of communicating consistency.

Before interpreting this finding, it is important to mention an alternative explanation that could be responsible for the effect. The effectiveness text module was by far the longest module so that a “mere exposure to more information” effect (cf. reference [42] for the general effect and reference [15] for the effect in RF-EMF risk perception) on risk perception cannot be ruled out with our design. None-the-less, we think that the content, rather than magnitude, of the message are more likely to explain our findings. Specifically, in the effectiveness condition we explained how effective precautionary measures are by providing two important facts about individual RF-EMF exposure; that exposure declines exponentially with the distance to the RF-EMF source, and that that mobile phones generally contribute more to the individual exposure to RF-EMFs than base stations do. While this information about the exposure patterns was intended to explain the effectiveness of the measures, the majority of the recipients might have focused on the information about the RF-EMF exposure patterns themselves, neglecting the line of argumentation the information was embedded in; in this case explaining the effectiveness of the precautionary measures. Informing about exposure patterns has been shown to selectively increase the risk perception of mobile phones and decrease the risk perception of base stations [15]. This is also in line with other research that has shown that RF-EMF risk perception is highly correlated with RF-EMF exposure perception [43]. Our results suggest that on the group level, the information about exposure patterns—presumably regardless of whether it is an explanation for the measures’ effectiveness or not—shifts the recipients’ focus to the mobile phone as a major RF-EMF source and hence the risk perception “while talking on the phone” is increased.

The results demonstrate that message interpretation is an activity of the recipient that is not under the control of the communicator. Therefore, even additional explanations that serve in the view of the communicator a strict reassuring goal, may be regarded as alarming by the recipient.

Finally, we would like to discuss our dependent variables. While conditional risk perception measures have been known in risk perception research [29,30] they have not been used in previous studies about RF-EMF precautionary communication. We derive from our data that their use is promising and might help uncover new facets of people’s risk perception in relation to precaution. Of particular importance is the fact that the mean of unconditional risk perception was lower than the mean of conditional risk perception if no precautions are applied. We interpret this as an indication that (some) people already include the application of precautionary measures or the potential to apply them in their unconditional ratings. Furthermore, conditional risk perception if precautions are taken had a mean of around 3 on a seven-point Likert scale in every group. This indicates that even if precautions are taken people do not let go of all their concerns. However, two potential problems regarding the conditional measures need mentioning. On the one hand, the conditional risk measures might have confounded our experimental groups; mentioning precautionary measures in the wording of the question might itself trigger the same effect that the precautionary text module does. There is, however, no way around this when assessing conditional risk perception. Besides, the unconditional risk perception measure was assessed prior to the conditional ones and is hence in no way influenced by them. On the other hand, the conditional risk perception measures might be providing cues for our subjects [44] in the sense that the questions shape the answers and therefore confound how much a person’s risk perception depends on the application of precautionary measures. We tried to mitigate this potential issue by introducing the two questions with the statement that “some people might answer both questions differently while others might give the same answer”. Future research could usefully validate this form of measurement in the context of RF-EMF precautions.

## 5. Conclusions

All in all, this study with an Australian population quota sample came to three major findings. Firstly, information about precautionary measures did not increase the risk perception of RF-EMFs during mobile phone calls in comparison to a group that had received information about the current limits. In previous studies, this effect had been detectable. Why this effect was absent remains an open question.

Secondly, the additional information that we added to reduce risk perceptions did not achieve the intended effect. Making the message more consistent for the reader did not have any effect on the mean level, and explaining the effectiveness of the precautions even led to an increase in risk perception scores. There might be other ways of amending precautionary messages in order to reduce risk perception, but these two ways do not seem to work.

Thirdly, from a methodological perspective, the conditional measurement we implemented in this study seems to be a promising avenue for future studies in the field of precautionary communication. However, this first attempt of a differentiation in this area should not be over-interpreted as the conditional measurement needs to be validated in future studies.

## Figures and Tables

**Table 1:** Different types of messages about radiofrequency electromagnetic fields (RF-EMFs) from mobile phones in the different study groups.

Group No.	N	Information Group	Gender % Female	Age Mean (SD)
1	303	No text group ^1^	53.8	42.7 (13.2)
2	281	Basic group	53.7	43.8 (13.2)
3	279	Precaution group	53.4	43.4 (13.9)
4	297	Consistency group	51.2	42.5 (13.5)
5	292	Effectiveness group	56.5	42.8 (13.8)
6	265	Consistency + effectiveness group	56.2	43.1 (13)

^1^ In Group 1, participants received an introductory sentence before answering the conditional risk perception questions. The sentence stated that some people apply precautionary measures. Without this information, the questions would have been meaningless to those who had never heard about precautionary measures before.

**Table 2:** Structure of the conducted analyses of variance.

Hypothesis No.	Factor	Factor Levels
Hypothesis 1 and 2	Information Type	no text; basic text; precaution
Hypothesis 3, 4 and 5	Information Type	precaution; consistency; effectiveness; consistency + effectiveness

**Table 3:** Means, Standard Deviations and Correlations of the Risk Perception Variables (N = 1717).

	CR1 ^1^	CR2 ^2^	UR ^3^
**CR1**	4.65 (1.56)		
**CR2**	0.50	3.08 (1.35)	
**UR**	0.77	0.50	4.30 (1.44)

^1^ CR1 = Conditional risk perception without precautions; ^2^ CR2 = conditional risk perception with precautions; ^3^ UR = Unconditional risk perception. Means (standard deviations). All correlation coefficients are significant (*p* < 0.001).

**Table 4:** Means (99% confidence intervals) of risk perception in the experimental groups.

Experimental Group	UR ^1^	CR1 ^2^	CR2 ^3^
no text	4.07 (3.84–4.30)	4.30 (4.06–4.54)	3.08 (2.88–3.28)
basic text	4.21 (3.98–4.44)	4.51 (4.26–4.76)	3.19 (2.98–3.40)
basic + precaution	4.35 (4.14–4.56)	4.55 (4.32–4.78)	3.11 (2.91–3.32)
basic + precaution + consistency	4.20 (3.98–4.41)	4.49 (4.27–4.72)	3.01 (2.81–3.21)
basic + precaution + effectiveness	4.50 (4.29–4.72)	5.05 (4.83–5.28)	3.03 (2.83–3.24)
basic + precaution + consistency + effectiveness	4.49 (4.28–4.71)	5.03 (4.79–5.26)	3.08 (2.86–3.30)

^1^ UR = Unconditional risk perception; ^2^ CR1 = Conditional risk perception without precautions; ^3^ CR2 = conditional risk perception with precautions.

**Table 5:** Summary of hypotheses decisions.

Hypothesis No.		Specific Hypothesis for UR, CR1 and CR2 ^1^	Decision
Hypothesis 1	a	UR_precaution_ > UR_basic message_	Reject
	b	CR1_precaution_ > CR1_basic message_	Reject
	c	CR2_precaution_ ≤ CR2_basic message_	(Accept) ^2^
Hypothesis 2	a	UR_basic message_ > UR_no message_	Reject
	b	CR1_basic message_ > CR1_no message_	Reject
	c	CR2_basic message_ > CR2_no message_	Reject
Hypothesis 3	a	UR_precaution_ > UR_consistent precaution_	Reject
	b	CR1_precaution_ > CR1_consistent precaution_	Reject
	c	CR2_precaution_ = CR2_consistent precaution_	(Accept)
Hypothesis 4	a	UR_precaution_ > UR_effective precaution_	Reject
	b	CR1_precaution_ = CR1_effective precaution_	Reject (countervailing)
	c	CR2_precaution_ > CR2_effective precaution_	Reject
Hypothesis 5	a(1)	UR_effective + consistent precaution_ < UR_consistent precaution_	Reject
	a(2)	UR_effective + consistent precaution_ < UR_effective precaution_	Reject
	b(1)	CR1_effective + consistent precaution_ = CR1_consistent precaution_	Reject (countervailing)
	b(2)	CR1_effective + consistent precaution_ < CR1_effective precaution_	Reject
	c(1)	CR2_effective + consistent precaution_ < CR2_consistent precaution_	Reject
	c(2)	CR2_effective + consistent precaution_ = CR2_effective precaution_	(Accept)

^1^ UR = Unconditional risk perception, CR1 = Conditional risk perception without precautions, CR2 = Conditional risk perception with precautions; ^2^ “(Accept)” refers to results that were consistent with our predictions but for which the null hypothesis was not rejected.

## Appendix A

### Basic text module

The International Commission for Non-Ionizing Radiation Protection—a body collaborating with the World Health Organisation (WHO)—has recommended limits of exposure to electromagnetic fields emitted by mobile phones and mobile phone towers (the exposure is the strength of the electromagnetic field that reaches a person). Australia, like most other countries, has adopted these limits in its laws. A large number of studies have been performed to investigate whether mobile phone use poses a potential health risk. It is the assessment of the Australian Radiation Protection and Nuclear Safety Agency (ARPANSA) and other national and international health authorities, including the WHO, that there is no established scientific evidence that the use of mobile phones causes any health effects within these limits.

### Precaution text module

However, the possibility of harm cannot be completely ruled out. ARPANSA and other authorities recommend the following measures as highly effective means to minimize your personal overall exposure, and especially the exposure of your head:

− use a headset when talking on the mobile phone.
− limit the duration of mobile phone calls.
− make mobile phone calls where the reception is good. Your phone sends a weaker signal under good reception conditions.
− use a mobile phone with a low SAR value. The SAR value indicates how much electromagnetic energy your head absorbs when you make a phone call.
− use the mobile phone’s loudspeaker when talking on the phone.
− do not make calls with a mobile phone when a normal wired phone is available.
− send a text message (SMS, What’s App, Email or similar) instead of making a voice call.

### Consistency text module

**Why does ARPANSA communicate precautionary measures despite the safety of the existing limits?**

Despite much research, there has been no established scientific evidence for any health effects of electromagnetic fields within the limits implemented in Australian law. However, ARPANSA also provides information about precautionary measures. Although this may appear contradictory, it is provided for those people who may be concerned (regardless of the science), as a means of assisting them to reduce their exposure.

### Effectiveness text module

**How do the precautionary measures substantially reduce exposure?**

Electromagnetic fields decline very rapidly with increased distance from the source of the field

During phone calls, a mobile phone functions as an antenna and sends signals, just like mobile phone towers do. The most effective way to reduce exposure to electromagnetic fields during mobile communication is to increase the distance between the mobile phone and the user (e.g. by using a headset). People often underestimate the exposure reduction that can be achieved by having the mobile phone even a few centimetres further away from the head, but physics shows us that this reduction is very large. Therefore, using a wired headset or the loudspeaker of the phone reduces the exposure substantially, as it physically moves the exposure source away from the brain. The following table shows the reduction of exposure that can be achieved (as a percentage) by moving the phone away from the ear, compared with having the phone against the ear.

**Distance to Source of Electromagnetic Field**	**Exposure Reduction (compared with the standard exposure)**
Phone at ear (approximately 2.5 cm from the brain)	standard exposure to the brain (at this distance, exposure is within the limits regulated by law and not deemed harmful)
5 cm from ear	89% exposure reduction
10 cm from ear	96% exposure reduction
25 cm from ear	99% exposure reduction

During phone calls, mobile phones are responsible for most of the exposure, not mobile phone towers

Although a mobile phone tower may emit a strong signal, at the distance that mobile phone towers are generally from people these emissions are extremely small and far smaller than those from your mobile phone.

Increasing the distance to the phone (e.g. with a headset), limiting exposure time to it (e.g. via sending text messages, keeping phone calls short and using wired phones when available) or limiting the emissions of your phone (by buying a phone with a low SAR value or by restricting calls to situations with good reception) will lead to the reduction of the largest share of your overall exposure to electromagnetic fields from mobile telecommunication technologies.
